# Freeze all Policy: An Expanded Strategy Before Its Clinical Approval

**Published:** 2017

**Authors:** Mohammad Reza Sadeghi

**Affiliations:** Editor-in-chief

The introduction of vitrification method for cryopreservation of gametes/embryo in assissted reproductive technology (ART) increased the efficacy of freez-thawing method esspecislly its post-thawing survival rate. This efficient method provides an excellent solution to postpone embryo transfer in natural cycles with adequate number and timing for main challenges of ART such as polycystic ovarian syndrome (PCOS), pre-implantation genetics diagnosis/screening (PGD/PGS), advanced female age, low responders, repeated implantation failure (RIF), multiple pregnancy, third-party reproduction, *etc* (1).

The first baby was born from frozen-thawed embryos in Australia in 1984 and subsequently in USA in1986. Regarding limitations of cryopreservation techniques, its low efficiency and low post-thawing survival rate of embryos, less than 1% of all cycles were frozen-thawed embryo transfer (FET) at that time. It increased up to 30% in 2004 and this rate is growing not only due to its better success rate compared to fresh embryo transfer but also because of worldwide advocacy of elective single embryo transfer (eSET) over multiple embryo transfer. At recent, it is estimated that more than 50% of the children born following ART are from frozen-thawing cycles (2).

The success rate of IVF was less than 1% at the birth of Louise Brown, but its success rate has increased with the development of ART techniques up to 30% in 2000. The speed of growth has declined since then; however, the development of cryopreservation techniques leads to more success of frozen-thawing cycles than fresh ET, so that the overall success of frozen-thawing blastocyst transfer cycles is more than 50% and in some cases, the use of embryo transfer together with PGS techniques increase the ART success rate up to 80% (3, 1).

At recent, an efficient cryopreservation technique for gametes, embryos and reproductive tissues has important functions that are critical and the main pratice of each ART program. It extends time for embryo evaluation, allows systematic application of elective single embryo transfer policy, enables egg banking for donation and/or for oocyte accumulation, permits fertility preservation for medical and non-medical indications, enhances cumulative live birth rate per oocyte retrieval cycle and also provides the opportunity to perform cycle segmentation. Following the successful birth of the first IVF baby and low success rate of fresh ET, Edwards and Steptoe discussed the negative effects of supper physiological levels of exogenous and endogenous hormones during ovarian stimulation on endometrium and its receptivity. Therefore, they suggested that freezing all embryos for subsequent transfers of thawed embryos is the best method (3).

Unfortunately, the cryopreservation technology was very poor especially at early stages of its development, so the first IVF baby was born from a fresh ET and thus fresh ET became the default standard of ART. Over time, this approach has changed, so the rate of fresh ET reduced and in return the rate of frozen embryo transfer increased, so that the freeze all policy is going to be the dominant dialogue and practice in ART. Despite limited evidence, there is some concern on its effectiveness, increased cost-per-pregnancy and time-to-pregnancy (1). Similar to all medical procedures, in spite of several advantages and strengths of freeze all policy, it has some threats especially significantly increased rate of large-for-gestational age (*LGA*) *babies* in frozen cycles (4).

There are few randomized clinical trials (RCT) in comparing freeze all protocols with fresh embryo transfer; however, most studies are not randomized and also there are four acceptable RCT works in different groups of infertile couples which are difficult to be compared. According to these results, benefits of freeze all strategy are likely for patients with good ovarian response and women with suboptimal/poor response had better results with fresh ET. Moreover, obstetric and perinatal outcomes regarding frozen ET are controversial (5).

In addition, studies about cost and time concerns in freeze all policy do not seem to be valid since in some cases, the time spent and the cost per successful clinical pregnancy is less than that in fresh ET (6, 7).

Since the health of ART born children during the whole length of life is important, considering above concerns, caution should be taken in using freeze all policy universally for all infertile couples. Therefore, more evidence is needed on the ramifications of freezing embryos and until then ART programs might adopt an individualized policy. Future studies may confirm the advantages of FET or refuse these findings and support fresh ET. Therefore, clinicians should carefully decide before adopting freeze all embryos as a beneficent policy for all patients.
